# Wound healing effects and antibacterial properties of heterologous platelet-rich plasma on *Staphylococcus aureus* induced septic wounds in rabbits

**DOI:** 10.5455/javar.2022.i617

**Published:** 2022-09-30

**Authors:** Mst. Jakia Sultana, Mst. Antora Akter, Nelema Yesmin, Md. Azizul Haque, Marzia Rahman, Md. Mahmudul Alam

**Affiliations:** 1 Department of Surgery and Obstetrics, Faculty of Veterinary Science, Bangladesh Agricultural University, Mymensingh, Bangladesh; 2 Department of Microbiology and Hygiene, Faculty of Veterinary Science, Bangladesh Agricultural University, Mymensingh, Bangladesh; † These two authors contributed equally.

**Keywords:** Antibacterial activity, epithelialization, platelet-rich plasma gel, skin infection, wound healing

## Abstract

**Objective:**

This study has been designed to determine the effectiveness of heterologous platelet-rich plasma (hPRP) against infected wounds in rabbits.

**Materials and Methods:**

*Staphylococcus aureus* contamination was present in 24 surgical incisions, each 8 mm in diameter. The animals were then divided into two groups as follows: group A, also known as the hPRP group, received topically applied, freshly manufactured hPRP twice weekly, and group B, also known as the control group, only received sterile saline. Evaluations of the histological architecture of wounds, posttherapeutic morphology, morphometry, and *in-vitro* and *in-vivo* antimicrobial potentials of hPRP were made.

**Results:**

Rabbits that were given hPRP exhibited quicker rates of wound contraction and shorter healing times. The samples from day 7 in the hPRP group showed less inflammation and more structured fibroblasts than those from the control wounds, according to histological analysis. On day 21 of the histological examination, the hPRP group’s epidermis showed notable thickening. As demonstrated by *in-vitro* antibacterial activity, undiluted hPRP successfully suppressed *S. aureus* growth. A serum biochemical analysis showed that hPRP had no harmful effects on the liver or kidneys.

**Conclusions:**

Based on the findings of the histological features, antibacterial properties, and wound morphology, it can be said that hPRP gel holds promise as an alternative to antibiotics for the treatment of wound infections.

## Introduction

A wound breaks the skin’s natural anatomical structure and function. Chemical mediators, or cytokines associated with wound healing, increase and regulate the major cellular activities supporting healing [1]. This process involves numerous other proteins and growth factors, including platelet-derived growth factors, fibroblast growth factors, and epithelial growth factors [2]. A breakdown in this procedure may lead to wounds that take a long time to heal or extensive granulation tissue growth, which may stop the persistent inflammatory phase from progressing [3].

The prevalence of chronic degenerative diseases connected to infected wounds has significantly increased. Antibiotics have been overused to treat these types of wounds, which has led to the emergence of multidrug-resistant organisms that can result in life-threatening opportunistic infections [4]. Although therapies have made significant progress, there is a growing need for novel techniques to speed cutaneous septic wound healing. Platelet-rich plasma (PRP) might be an excellent alternative treatment in this situation.

PRP is a blood-derived substance with a large concentration of platelets and can release several growth factors and cytokines when activated. Due to its simplicity in preparation, high concentration of growth factors, and low immunogenicity, it has been extensively used in numerous postsurgical and medicinal therapies. In the area of wound healing, PRP has a range of potential therapeutic and experimental benefits [5,6]. PRP can produce larger quantities of vascular endothelial growth factor, which improves the vascularization of third-degree burns and can thus improve the prognosis of burn wounds [7]. Additionally, PRP has been demonstrated to generate many antimicrobial molecules, lessen local inflammation, and guard against wound infections [8].

PRP can be obtained from various sources, including heterologous, autologous, and donors. The benefit of using autologous supplies is that they reduce immune rejection reactions and spread infectious diseases [9]. The use of autologous PRP may be limited in specific circumstances, such as those related to cardiovascular disease, diabetes mellitus, hypertension, and severe burns. It would be interesting to investigate the utilization of additional PRP sources in these circumstances. Based on these findings, our study aimed to examine heterologous platelet-rich plasma (hPRPs) ability to repair wounds infected in rabbits.

## Material and Methods

### Animals

The Faculty of Veterinary Science of Bangladesh Agricultural University, Mymensingh’s Animal Welfare, Experimentation, and Ethics Committee, approved and provided guidelines for the animal experimentation that was conducted [approval number: AWEEC/BAU/2021 (46)]. This study used 12 New Zealand white rabbits with average body weights of 2.5 kg and ages ranging from 90 to 120 days (regardless of sex).

### Experimental design

On each side of the animals’ spinal column, 24 full-sized, open-circumferential cutaneous wounds were created. Two groups were studied with them. In the hPRP group or group A, 12 surgical lesions were produced in six animals that received 2 weekly local applications of fresh hPRP gel. In the control or group B, similar to group A, the animals in this group only received sterile phosphate buffered saline (PBS).

### Surgical technique

Following an overnight fast, all rabbits were dehydrated for 6 h. Ketamine hydrochloride (KETALAR^®^, Popular Pharmaceuticals, Bangladesh) and xylazine hydrochloride (XYLA^®^, Interchemie Werken, Holland) were used to induce anesthesia at doses of 5 and 35 mg/kg, respectively. The operating sites were surgically prepared. Then, while still protecting the muscle, two 8 mm diameter circular incisions were made along either side of the spinal column. *Staphylococc*us aureus 10^5^ colony-forming units (CFUs) containing 200 µl medium was applied to each wound immediately (day 0). The wounds were then covered with sterile vaseline gauze to induce sepsis. Tramadol hydrochloride (0.5 mg/kg, i/m) was administered to the animals twice daily for the first 24 h following surgery to deal with any initial discomfort.

### Preparation and activation of hPRP gel

hPRP was produced using bovine fresh total blood using a modified version of the method described by Badade et al. [10]. Briefly, whole blood (18 ml) was drawn from the bovine jugular vein into a disposable plastic syringe and transferred to a sterile plastic conical tube containing 2 ml of 3.8% sodium citrate as an anticoagulant. After proper mixing, the tube was centrifuged at 1,000 rpm for 13 min. As a result, the blood was split into its three basic components: an upper layer consisting of plasma and a few platelets; an intermediate layer comprising a white blood cell -rich buffy coat; and the bottom layer, mostly made up of red blood cell (RBCs). All the content above the RBCs zone were carefully pipetted and taken to an empty sterile tube of 10 ml capacity. This sample was again centrifuged at 2,000 rpm for 8 min. It produced a small red fraction at the bottom and a clear supernatant at the top of the tube. The upper two-thirds of the sample were labeled platelet-poor plasma (PPP) and the lower one-third was labeled PRP. The PPP was removed and PRP was collected. This newly created PRP was activated before use by adding 10% calcium chloride (55 µl/ml of PRP), and it was then left undisturbed for 25–30 min to allow for proper gel formation.

### Therapy

The bandage was changed after 72 h and hPRP gel therapy began. This continued for the next 2 weeks until the wound healed. The control group underwent the same treatment with sterile PBS.

### Morphometric assessment of wounds

Digital slide calipers were used to measure the wound contraction, which was then calculated and displayed as a percentage using the following formula:


$$\%\mathrm{Wound}\;\mathrm{Contraction}=\left\{\frac{\mathrm{Wound}\;\mathrm{area}\;\mathrm{at}\;\mathrm{day}\;0\;-\;\mathrm{Wound}\;\mathrm{area}\;\mathrm{at}\;\mathrm{day}(n)}{\mathrm{Wound}\;\mathrm{area}\;\mathrm{at}\;\mathrm{day}\;0}\right\}\times 100$$

where *n* = days 5, 7, 9, 11, 13, and 15.

### Histopathological evaluation and assessment of re-epithelialization

Wound biopsies were taken from the rabbits under general anesthesia on days 7, 14, and 18 following wounding. After being formalin-fixed for 24 h, tissues were paraffin-embedded. Hematoxylin and eosin were used to stain sections that were 5 µm thick. The slides were then examined using a photographic microscope (MICROS^®^, Austria). Using Image-J software, images at a greater magnification (×400) were utilized for epithelial thickness measurement to assess re-epithelialization (NIH and University of Wisconsin, USA).

### Antibacterial evaluation

The wound swab samples were collected on days 4, 9, and 13 following surgery. The nutrient agar media were first inoculated, followed by 100 µl of mannitol salt agar media, and the samples were incubated overnight at 37°C. The *Staphylococcus* spp. load was determined by multiplying the total number of the species by CFU/ml. Utilizing the microtiter plate method, antimicrobial activity was assessed *in vitro.* The microtiter plate spent the night being incubated at 37°C. By observing changes in the media’s color, hPRP’s antibacterial activity was evaluated.

### Serum biochemical tests

A semi-automatic biochemistry analyzer (CLINDIAG^©^, UK) was used for the biochemical assays of Total Protein, Alanine Transaminase (ALT), and Aspartate Aminotransferase (AST).

### Statistical analysis

Statistical analyses were carried out using Student’s *t*-test or independent sample t-test using the Statistical Package for the Social Sciences version 20.0. Data were displayed as mean and standard error of the mean. Probability *p* < 0.05 or less was considered statistically significant.

## Results

### Morphometric analysis

**Figure 1.**
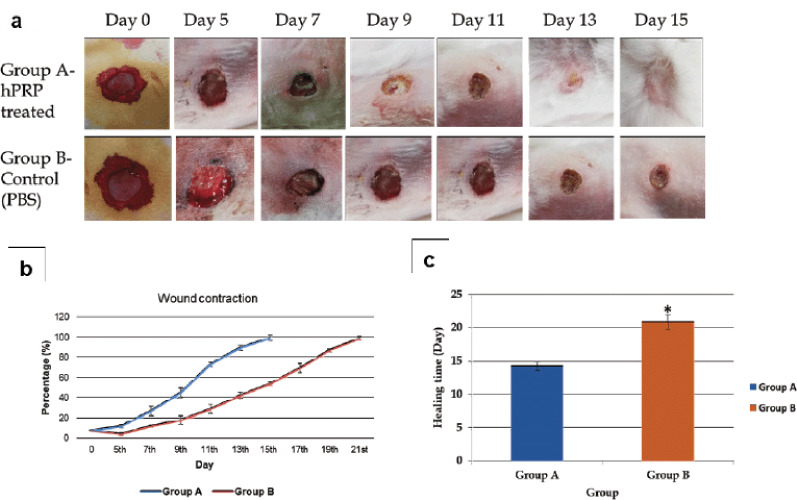
Progress of wound healing. (a) Macroscopic evolution of wound contraction process in both the groups at different time points of the experiment. (b) Percentage of wound contraction. (c) Complete healing period (Mean value). * indicates significant difference at 5% level.

Figure 1a shows how the healing process developed over time. A thorough analysis of the data revealed notable differences between the animal groups used in this study. The wound contraction in group A was considerably (*p <* 0.05) faster than that in group B at each time point indicated (Fig. 1b). On day 5, the wound treated with PBS had a mean contraction percentage of 4.52 ± 1.28 mm, while the average for the groups treated with hPRP was 12.39 ± 1.67 mm. After 2 weeks of wound expansion, there was a significant (*p <* 0.01) difference between the groups (89.95 ± 2.76 mm versus 42.5 ± 3.22 mm in hPRP versus the control group, respectively). In the hPRP-treated wound, the whole contraction occurred after 15 days, but it took 21 days in the saline-treated control group (Fig. 1c).

### Histological assessment

**Figure 2.**
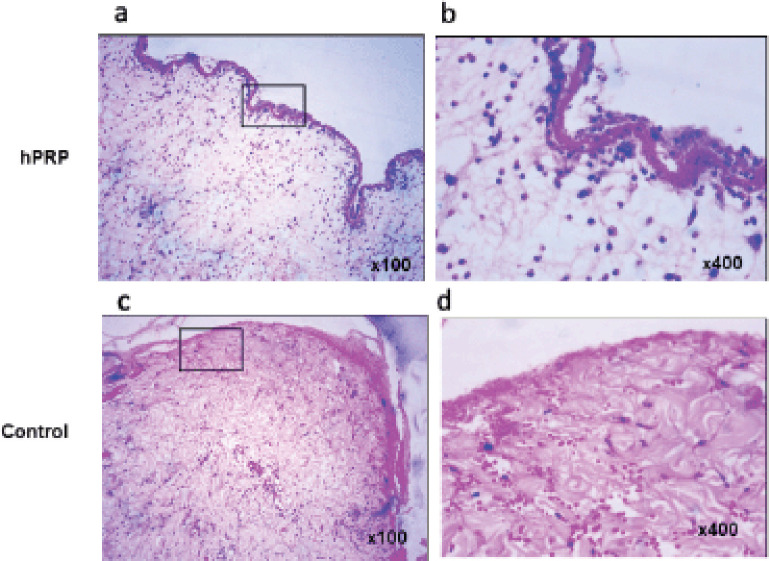
Hematoxylin and eosin-stained biopsies collected on day 7 postwounding. (a and b) The epithelialization was pronounced and continuous. Wounds treated with hPRP showed fibroblasts and freshly produced collagen bundles. (c and d) In the control group, the epithelial lining was disturbed, collagen was reduced, and macrophages remained in the stroma. Images in the right panel are magnified from the box of the left panel.

**Figure 3.**
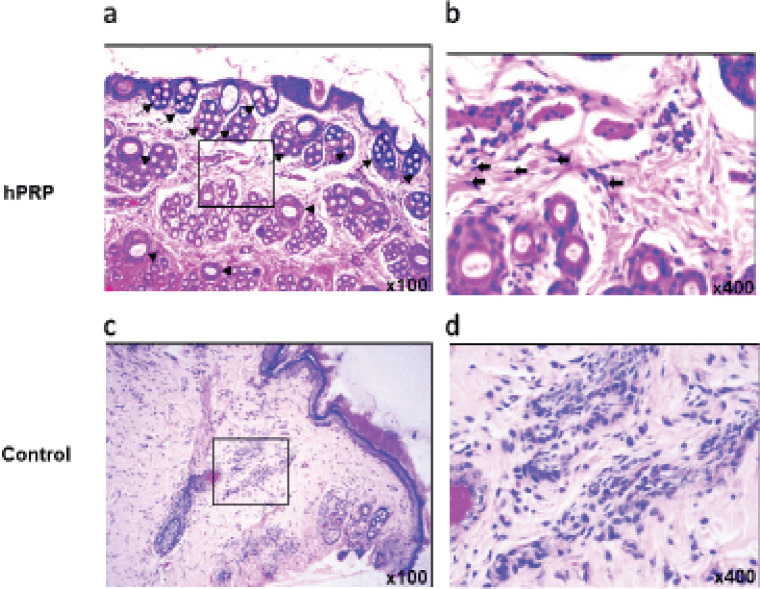
Histopathological analysis of the skin wound tissues on day 14 postsurgery. (a) On day 14, neovascularization (arrowhead) and abundant proliferation of hair follicles (small arrow) were evident. (b) Numerous orderly arranged fibroblasts (large arrow) were seen in the hPRP-treated group, which accumulated between condensed collagen bundles. This group demonstrated a decrease in the number of reactive cells and tissue response. (c and d) In the control group, wound stroma included hemorrhage and loose and disorganized collagen with a large number of reactive cells. Images in the right panel are magnified from the box of the left panel.

Hemorrhagic fibrin clots (scabs) were accumulated in the wound gaps on day 7 of the healing process, signifying the end of the inflammatory phase (Fig. 2a and b). On day 7 of the experiment, neutrophils began to penetrate the injured tissues. The majority of inflammatory cells in hPRP rabbits are macrophages and lymphocytes. The hPRP-treated wound surfaces (Fig. 2a and b) appeared to be fully epithelialized compared to the control wounds (Fig. 2c and d). The collagen organization in wounds treated with hPRP appeared denser and more organized. On day 14, the histological study of the wounds treated with hPRP showed a significant improvement in the healing process, with almost entirely united wound boundaries and better granulation tissue (Fig. 3a and b). Compared to control wounds, hPRP-treated wounds had different fibroblast counts and appearances. We observed significant growth of hair follicles in the testing area of treated groups with hPRP (Fig. 3a and b), indicating sound and effective healing. This trait was conspicuously absent from the wounds of the control group (Fig. 3c and d). On day 21, the hPRP-treated lesions were epithelialized entirely, as we had seen.

**Figure 4.**
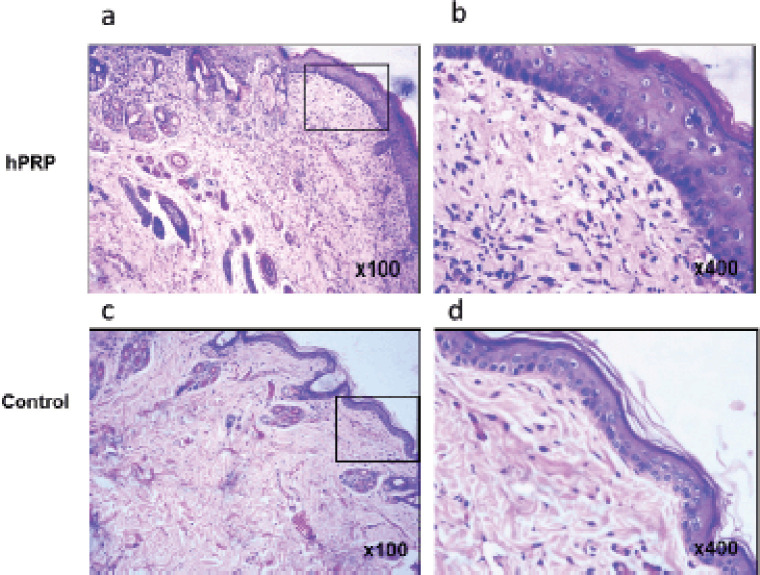
Photomicrographs of hematoxylin and eosin-stained histological sections on day 21. (a and b) Images show complete epithelialization and keratinization. In hPRP gel wounds, there were structured interconnecting collagen fibers and a discrete infiltration of inflammatory cells. (c and d) On day 21 postwounding, the control group’s wound had a much weaker epithelium covering and keratin layer, flabby connective tissue, and irregularly arranged collagen fibers. Images in the right panel are magnified from the box of the left panel.

Fresh collagen bundles were squeezed into the wound’s stroma (Fig. 4a and b). In the first 2 weeks after the incision was made, the number of newly produced capillaries increased dramatically, and it did not start to decline until the third week of the healing process. These characteristics significantly differed from the control groups (Fig. 4c and d).

### Measurement of re-epithelialization

**Figure 5.**
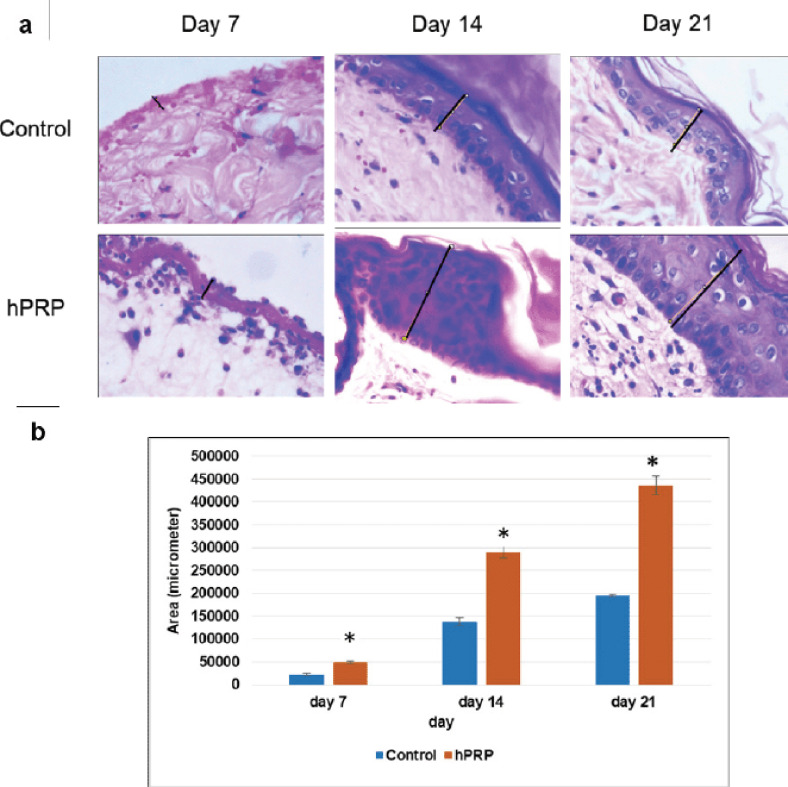
Quantitative assessment of re-epithelialization of wounds treated with hPRP and PBS (control). (a) Representative higher magnification images (×400) of day 7, 14, and 21 were selected to measure full-thickness epithelium with the help of Image-J software. (b) Data were analyzed through an independent sample *t*-test and shown in the bar graph. * indicates *p* < 0.05.

Our results showed significant differences (*p* < 0.01) in the epithelial layer thickness covering the wound surface on various healing days: 49,918.93 ± 2,648.47, 289,718.7 ± 12,038.54, 436,033.3 ± 20,820.05 µm against 22,224.9 ± 2,603.49, 137,496 ± 9,935.1, 195,439.7 ± 2,971.27 µm in hPRP and control wounds, respectively (Fig. 5a and b).

### In-vivo antibacterial efficacy

**Figure 6.**
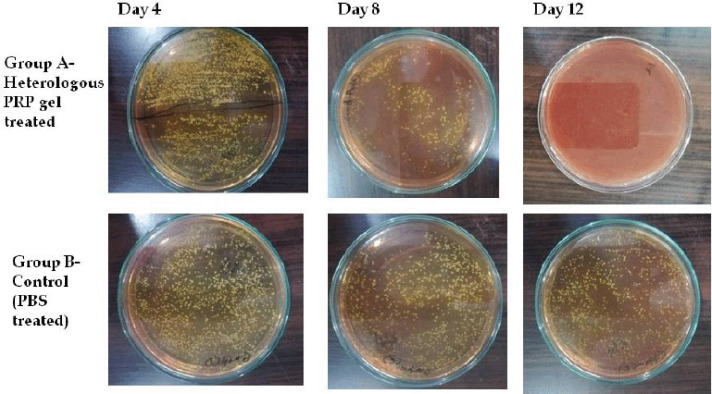
Determination of the bacterial load in the wounds of rabbits on different days of treatment.

As shown in Figure 6 and Table 2, the staphylococcal loads in the hPRP-treated group rapidly decreased on day 4 after wounding and infection, but there were no changes in the PBS-treated (control) wounds. The hPRP-treated group did not grow S. aureus on day 12, but the control group experienced significant bacterial growth (4.1105 CFU/ml).

### In-vitro antibacterial activity of hPRP

**Figure 7.**
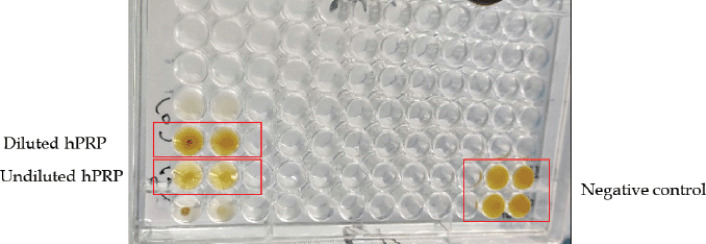
*In-vitro* antibacterial activity of hPRP in a 96-well plate. Changes in the color of the media indicate inhibition of bacterial growth.

Microdilution analysis was used to investigate the *in-vitro* antibacterial activity of hPRP. In the well containing freshly generated hPRP without dilution, *S. aureus* did not grow, demonstrating that the bacterial growth was inhibited at the original dose. However, no antibacterial activity was observed in the wells containing diluted hPRP which was almost similar to the untreated negative control (Fig. 7).

### Serum biochemical variables

We examined specific serum enzymes to see if there were any alterations in these biomarkers as the healing of the lesion advanced, possibly pointing to any adverse effects of hPRP on the body. TP levels were observed to be considerably (*p <* 0.05) higher in the control group than in the animals that had received hPRP treatment. AST and ALT are two crucial indicators for assessing the liver’s functioning state. In the hPRP-treated animals, both enzymes were within the normal range. At the same time, they were significantly raised in the control group (Table 2), demonstrating that hPRP had no adverse effects on the liver or kidney.

## Discussion

In this study, we looked at the clinical efficacy of topically applied hPRP on rabbits with septic wounds. Ultimately, we observed the enhanced function of this biomaterial in the healing process. Our results in terms of healing time were quite good due to hPRP-prompted wound healing (15 versus 21 days in the hPRP and control groups, respectively). In contrast to our findings, Ostvar et al. [2] found that the PRP and control groups healed in 17 and 25 days, respectively. In the hPRP-treated group, we observed quicker wound contraction; 99.59% contraction was attained in 15 days. This outcome demonstrated the hPRP’s faster wound healing abilities.

According to the histopathological investigation’s findings, several healing indices have undergone significant modifications. The exceptional epithelialization and precocious neovascularization caused by topical hPRP injection were thus followed by a deliberate collagen restoration phase. PRP skin infiltration stimulates the growth of more complicated granulation tissue and hastens the healing of wounds in dogs [11].

Our research day 7 of recovery showed that hPRP is biologically effective and has great potential to speed up the healing process by attracting significant numbers of polymorphonuclear cells. This might speed up the rate of angiogenesis. The use of PRP expedites the formation of new capillary loops in the dermis of rabbit models of wound recovery, according to Ostvar et al. [2]. In previous work, Leal et al. [12] demonstrated that inflammatory cells enter skin wounds as they heal due to the debridement procedure. In turn, the inflammatory cells transport macrophages, which quicken the debridement process.

When we looked into altering wound fibroblasts, we discovered that hPRP-treated wounds displayed enhanced results on days 14 and 21 of healing. In their study, Golebiewska and Poole [13] discussed the potential causes, including the part that certain growth factors play in promoting fibroblast differentiation and collagen formation. Inside the stroma of wounds treated with hPRP, we have noticed significant dermal papilla regrowth, which we believe is related to the restoration of hair follicles. Our findings concur with those of Badis and Omar [14]. In another investigation, they found that applying PRP topically to sheep greatly enhanced healing, leading, among other things, to the development of hair follicles.

Our *in-vivo* and *in-vitro* hPRP antibacterial trials were a great success. Following topical application of hPRP gel to the resulting septic lesions, the CFUs of *S. aureus* significantly decreased (*p* < 0.05), demonstrating PRP’s antibacterial capabilities. Our results support earlier studies which found that hPRP gel dramatically lowers the number of germs in wounds as they heal [15]. The *in-vitro* antibacterial study’s findings showed that hPRP could reduce *S. aureus* at its starting concentration, proving that it could be undiluted to treat staphylococcal skin infections. Superoxide, hydrogen peroxide, and hydroxyl free radicals, also known to have antibacterial effects, are among the oxygen metabolites platelets that have been shown to be created. However, the precise mechanism by which PRP exerts these effects is still being studied [16]. To eliminate protozoal invaders, platelets can also bind, agglomerate, and internalize germs and engage in antibody-dependent cell cytotoxicity. Additionally, platelets secrete a large number of strong antibacterial chemicals [17].

We assessed a few serum biochemical enzymes in both groups on day 10 postwounding. In this study, the control group’s TP value was significantly higher than that of the hPRP group. In line with our findings, Handoo et al. [18] discovered that the treatment group had lower serum albumin levels than the control group.

The most frequent and accurate indicators of hepatocellular activity are AST and ALT blood levels. While the hPRP group’s AST and ALT values remained within the physiological range throughout the research, they substantially increased in the control group. This concurs with the conclusions reached by Handoo et al. [18]. This suggests that hPRP gel has no harmful effects on hepatocellular function.

**Table 1.** Quantitative analysis of *S. aureus* in the wounds of rabbits treated with hPRP gel (group A) and control (group B) on different days of treatment.

Days	Group A (CFU/ml)	Group B (CFU/ml)
4	7.8 × 10^5^	8.4 × 10^5^
8	3.5 × 10^5^	6.75 × 10^5^
12	0 (No growth)	4.1 × 10^5^

**Table 2.** Dynamics of serum biochemical parameters in response to hPRP treatment on day 10 postwounding in rabbits.

Parameters	Group A	Group B
TP (gm/dl)	3.93 ± 0.02^a^	4.62 ± 0.02^b^
ALT (IU/l)	4.68 ± 0.01^a^	5.25 ± 0.02^b^
AST (IU/l)	12.85 ± 0.02^a^	15.00 ± 1.00^b^

Values with a different superscript letters in the same row differ significantly (*p* < 0.05).

## Concslusion

Based on the results of wound morphological features, histological features, and antibacterial efficiency, it is feasible to conclude that hPRP gel may be a promising antibiotic alternative. But it is also essential to carry out lengthy in-vitro tests to look at the biomechanical characteristics and degradation pattern of hPRP. More clinical trials are also needed to evaluate the potential application of hPRP in tissue engineering and epidermal regeneration.

## List of abbreviations

mm, millimeter; h, hour; mg, milligram; kg, kilogram; µl, microliter; µm, micrometer; ml, milliliter; %, percentage; rpm, rotation per minute; min, minutes; RBC, red blood cell; °C, degree Celsius; *S. aureus*, *Staphylococcus aureus*; spp., species.
